# Neglected tropical diseases in Yemen: a systematic review of epidemiology and public health challenges

**DOI:** 10.1186/s12889-025-21700-z

**Published:** 2025-02-07

**Authors:** Ibrahim Ahmed Ahmed Alhothily, Rahmat Dapari, Nazri Che Dom

**Affiliations:** 1 Centre of Environmental Health & Safety, Faculty of Health Sciences, Universiti Teknologi MARA (UiTM), UITM Cawangan Selangor, Puncak Alam, Selangor 42300 Malaysia; 2 Department of Community Health, Faculty of Medicine and Health Sciences, Universiti Putra Malaysia, Serdang, 43400 Malaysia; 3 Integrated Dengue Research and Development, Faculty of Medicine and Health Sciences, Universiti Putra Malaysia, Serdang, 43400 Malaysia; 4 Integrated Mosquito Research Group (I-MeRGe), Universiti Teknologi MARA (UiTM), UITM Cawangan Selangor, Puncak Alam, Selangor 42300 Malaysia; 5 Institute for Bioaffiliationersity and Sustainable Development (IBSD), Universiti Teknologi MARA, Shah Alam, Malaysia

**Keywords:** Neglected tropical diseases, Yemen, Epidemiology, Dengue, Sanitation, Vector control, Conflict

## Abstract

**Background:**

Yemen has experienced a dramatic increase in neglected tropical diseases (NTDs) amidst ongoing conflict and humanitarian crises. This systematic review aims to consolidate and analyse the available literature on NTDs in Yemen, focusing on aetiology, geographic distribution, and associated risk factors.

**Methods:**

A comprehensive literature search was conducted across five international databases and one national database, resulting in 3,652 identified records. After screening and applying eligibility criteria, 230 articles were included in this review. Data extraction focused on publication year, study design, sample types, diagnostic methods, reported pathogens, and geographic distribution. The aetiology of reported NTDs was categorized into four groups: viruses, bacteria, protozoa, and helminths.

**Results:**

Viral NTDs were the most frequently reported, accounting for 39% of the articles, followed by bacterial (26%), helminthic (21%), and protozoal NTDs (15%). Dengue virus, hepatitis B and C viruses were the most prominent viral pathogens, while bacterial NTDs were primarily caused by *Escherichia coli*, cholera, and *Salmonella*. Schistosomiasis and ascariasis were the most reported helminth infections, whereas leishmaniasis and malaria were the leading protozoal NTDs. Geographically, over 69% of the reported studies focused on northern Yemen, with the highest concentrations in Sana’a, Al Hudaydah, and Taiz. The review identified multiple risk factors, including poor sanitation, inadequate water quality, and urbanization, exacerbating NTD prevalence.

**Conclusions:**

The findings highlight the significant burden and regional disparities of NTDs in Yemen, emphasizing the need for targeted interventions. Prioritizing improvements in sanitation, water quality, and vector control measures, alongside community engagement, is critical. Policymakers must allocate resources effectively to address the root causes of NTDs and strengthen Yemen’s healthcare infrastructure.

## Introduction

Neglected tropical diseases (NTDs) are a group of infectious diseases primarily affecting impoverished and marginalized populations in tropical and subtropical regions. These diseases are caused by various pathogens such as viruses, bacteria, protozoa, and helminths [1, 2]. Examples include malaria, dengue fever, schistosomiasis, leishmaniasis, and lymphatic filariasis. The transmission of NTDs is influenced by factors such as environmental conditions, including climatic changes and poor sanitation, as well as socioeconomic factors like poverty, inadequate healthcare infrastructure, and low levels of education. Despite their significant public health impact, NTDs often receive limited attention and resources, perpetuating cycles of disease and poverty [3]. Globally, NTDs affect over 1.7 billion people annually, disproportionately impacting impoverished communities in tropical and subtropical regions. These diseases contribute significantly to morbidity and mortality, with conditions such as malaria causing approximately 619,000 deaths in 2021, while dengue fever results in an estimated 390 million infections each year [4, 5].

Since 2015, Yemen has faced a devastating conflict, marked by Saudi-Emirate coalition intervention [6]. This protracted war has led to one of the world’s most severe humanitarian crises, inflicting immense physical destruction and crippling Yemen’s already fragile healthcare system [7]. In Yemen, the burden of NTDs is particularly alarming. For instance, malaria remains endemic in parts of the country, with an estimated 110,000 cases reported in 2022 alone [8]. Similarly, dengue fever outbreaks have surged in recent years, with thousands of cases reported annually [9, 10]. Cholera, another significant NTD, has affected millions in Yemen since the onset of the conflict, with the 2017 outbreak alone resulting in over 1 million suspected cases. Schistosomiasis, a waterborne parasitic disease, also continues to affect rural populations, particularly in areas with poor water and sanitation infrastructure [11, 12]. The displacement of over 2.4 million Yemenis due to the conflict has led to overcrowded, unsanitary living conditions in camps and host communities [13, 14]. These conditions significantly increase the risk of disease outbreaks, particularly preventable diseases such as NTDs, including malaria, dengue fever, cholera, and schistosomiasis. These diseases disproportionately affect displaced populations and communities with limited access to clean water, sanitation, and healthcare, compounding the humanitarian emergency.

In addition to the impacts of conflict, several other factors contribute to the spread of NTDs in Yemen. Climatic changes, such as rising temperatures and changing rainfall patterns, have created favourable conditions for vectors like mosquitoes and snails, increasing the risk of vector-borne diseases such as dengue and schistosomiasis [15]. Geographical changes, including urbanization and population displacement, have further exacerbated transmission dynamics by increasing human-vector contact [16]. Economic collapse, food insecurity, and widespread poverty have also strained public health systems, limiting access to essential prevention and treatment services [13, 14]. Behavioural and educational factors, such as low health literacy and poor hygiene practices, compound these challenges by hindering the effectiveness of public health interventions. Overcrowded and unsanitary living conditions in displacement camps and host communities have created ideal environments for the spread of NTDs and other communicable diseases. The collapse of water, sanitation, and hygiene (WASH) systems further heightens the risk of disease transmission [17, 18]. Despite the efforts of international organizations, including the World Health Organization and non-governmental organizations, interventions have been insufficient to curb the rise in NTDs [19]. The combined effects of these factors highlight the complexity of addressing NTDs in Yemen, necessitating a multifaceted approach that considers both direct and indirect contributors to disease transmission.

Addressing NTDs in Yemen requires identifying and leveraging lessons from successful interventions in similar settings. For instance, integrating mobile health units and low-cost vector control measures, such as insecticide-treated bed nets and targeted larval source management, has proven effective in comparable crises [20]. Moreover, local stakeholders such as community leaders, NGOs, and local governments must collaborate to ensure culturally sensitive implementation of these measures. While extensive documentation exists on the health impacts of Yemen’s war, comprehensive studies focusing on the epidemiology and burden of NTDs in this unique context remain scarce. Current research also lacks specificity in analysing indirect contributors, such as the interplay between climate variability, geographical shifts, and socioeconomic factors, on the spread of NTDs. Data-driven insights into these interactions are essential for guiding targeted interventions. A detailed understanding of these factors is urgently needed to inform more effective strategies. This includes designing feasible interventions tailored to Yemen’s conflict setting, such as implementing low-resource WASH solutions, training local health workers, and employing innovative vector control technologies. Enhancing community engagement through culturally appropriate education campaigns is vital for ensuring the sustainability of these interventions. By addressing these gaps, stakeholders can combat the burden of NTDs while building a resilient healthcare system in post-conflict Yemen. Ultimately, improving the control and prevention of NTDs will play a critical role in alleviating suffering and rebuilding the health of the Yemeni population. Therefore, the aim of this systematic review was to consolidate and analyse available information on NTDs reported in Yemen.

## Methods

### Criteria for pathogen selection: focus on infectious NTDs

The review focused on NTDs of infectious origin as recognized by the World Health Organization (WHO), U.S. Centers for Disease Control and Prevention (CDC), and *PLoS Neglected Tropical Diseases*. These sources were selected due to their authoritative roles in the global classification and management of NTDs. Non-infectious NTDs, such as snake bites, were deliberately excluded from this review to maintain a clear focus on diseases with infectious aetiologies. While snake bites are a significant public health concern in many tropical regions, their non-infectious nature does not align with the primary objective of this review. By narrowing the scope to infectious NTDs, the review ensures consistency and alignment with its overarching goal of understanding the epidemiology and transmission dynamics of infectious NTDs in conflict settings, particularly Yemen.

### Literature search strategy

A comprehensive literature search was conducted to address the following research questions: (1) What is the prevalence and distribution of NTDs in Yemen, particularly amidst the ongoing conflict? (2) How has the disruption of healthcare, sanitation, and displacement of populations affected the transmission dynamics of NTDs? and (3) What interventions have been reported in addressing NTDs in conflict settings, and how effective have they been? The search was carried out across multiple international databases, including PubMed, Google Scholar, Ovid Embase, Web of Science Core Collection, and Ovid Global Health. Additionally, a national database containing grey literature, such as locally published papers, conference presentations, and dissertations, was incorporated to ensure comprehensive coverage of evidence. The search was last updated on March 17, 2024, to include the most recent data.

The search strategy employed a combination of controlled vocabulary (e.g., MeSH terms in PubMed) and free-text keywords related to NTDs, conflict, and displacement in Yemen. For PubMed, the search included a combination of [Title/Abstract] and [MeSH] terms, while for Scopus, the search utilized [TITLE-ABS-KEY]. Specific keywords included “NTD,” “Yemen,” “conflict,” “displacement,” “water and sanitation,” and “interventions.” Boolean operators (AND, OR) were applied to refine the results. Efforts were made to align the search strategies across databases to minimize discrepancies; however, differences in indexing systems and search functionalities may have resulted in some variability. To ensure comprehensive coverage, no restrictions were applied to study design or publication period. In addition to peer-reviewed articles, grey literature, including locally published papers, conference presentations, and dissertations, was also incorporated to ensure comprehensive coverage of evidence. This approach facilitated the inclusion of diverse sources of information, particularly those reflecting local contexts and perspectives that may be underrepresented in international databases.

While the inclusion of grey literature is a significant strength, it is important to acknowledge its potential limitations. Grey literature may vary in quality, as it often lacks the rigorous peer review process associated with traditional academic publications. This can result in inconsistencies in methodology, reporting standards, and reliability of findings. To address these concerns, grey literature sources were critically appraised for relevance, methodological transparency, and alignment with the review’s objectives before inclusion. Despite these challenges, incorporating grey literature enriched the dataset by providing insights into local health interventions, outcomes, and challenges that might not otherwise be captured in peer-reviewed studies. This method enabled a broad yet targeted selection of studies, ensuring representation from both local and international contexts. By integrating both peer-reviewed and grey literature, the review sought to provide a more holistic understanding of NTD dynamics in Yemen, while maintaining transparency about the strengths and limitations of its data sources.

### Study selection and full text review

**Fig. 1:**
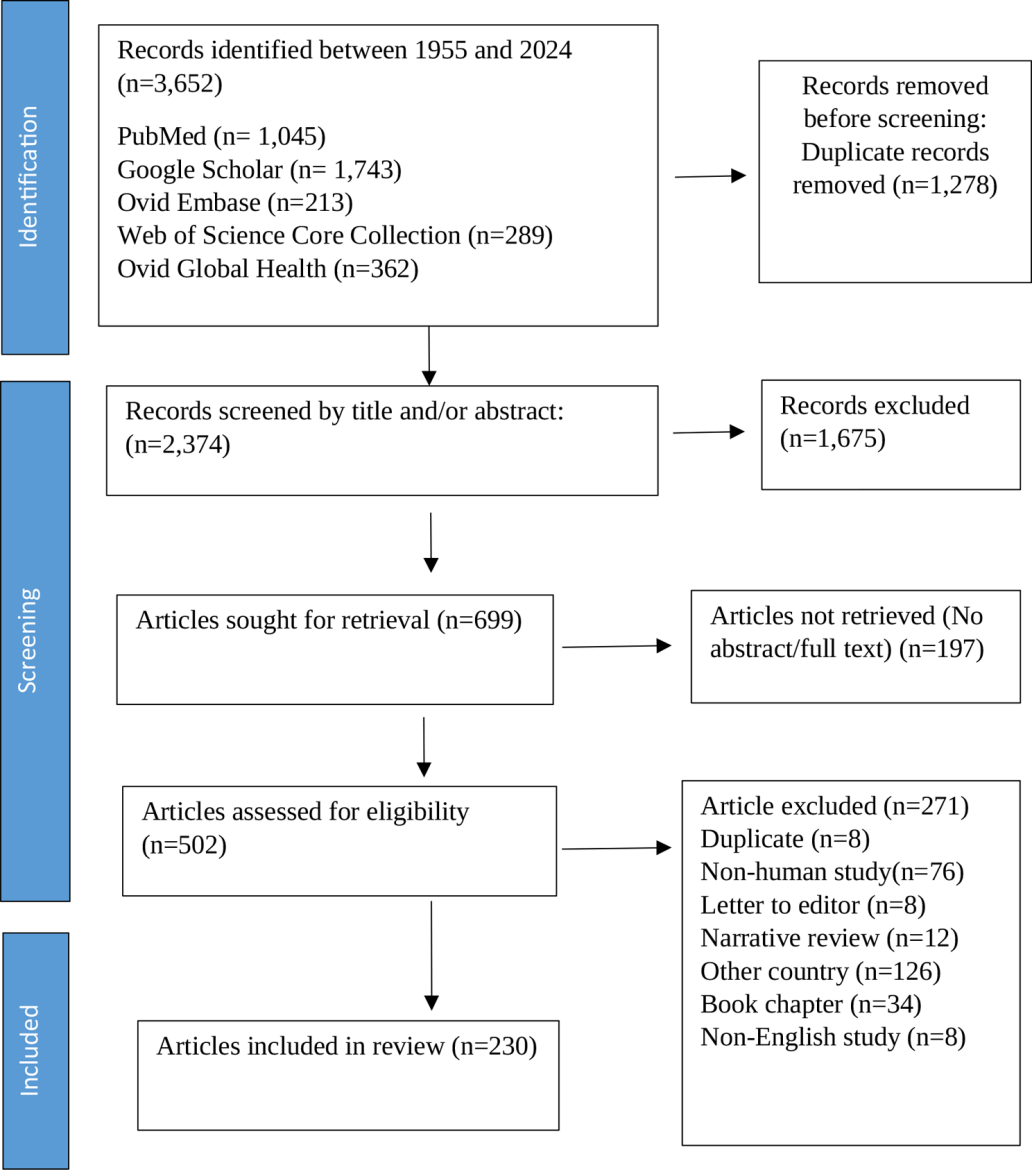
Flow chart of articles selection using PRISMA guidelines

The researcher independently screened search results, reviewing all titles and abstracts. Full-text articles for published studies and conference presentation abstracts were then assessed for eligibility based on pre-defined criteria. Eligible studies included observational studies, intervention trials, outbreak reports, case studies, and series involving human populations. Excluded studies included editorials, qualitative research, environmental surveys, non-human studies, and those focusing on snake bites. Articles not meeting eligibility criteria were excluded, with exclusion reasons systematically recorded. This process ensured a rigorous and transparent selection approach, detailed in the PRISMA flow diagram, which shows that out of 3,652 identified records, 1,278 duplicates were removed, 2,374 titles and abstracts were screened, and 230 articles were ultimately included in the review (Fig. 1).

### Data extraction

A standardized spreadsheet was used to ensure consistent and complete data extraction. Key variables extracted included publication year, first author’s name, study period, participants’ age groups, study location, and study design. Additional details such as study setting, sample types, pathogens, diagnostic methods, and the number of individuals tested were also recorded. Studies were classified into five categories: observational, case reports/series, outbreak investigations, surveillance, and cross-sectional studies.

To facilitate a comprehensive analysis, the study population was divided into four age groups based on standardized definitions: Infants and Young Children (newborns to 59 months, aligned with the WHO classification for early childhood), School-age Children (6 to 17 years old, consistent with UNICEF and educational age group classifications), Adults (18 to 64 years old, following international demographic standards), and Older Adults (65 years and above). In addition, the “Life Stage” category was used to encompass all age groups within a community, referred to as “Age Cohorts.” These definitions were applied consistently across studies to ensure comparability of findings and minimize ambiguity in age group categorizations. Studies were geographically organized by Yemen’s administrative divisions, enabling detailed analysis of research distribution across regions. The reported pathogens and diseases were grouped into four main categories: Viruses (Dengue Fever, Hepatitis B virus, Hepatitis C virus, Covid-19, Respiratory infection, Chikungunya, Human immunodeficiency virus, Cytomegalovirus, Rotavirus infections, West Nile virus, Rift Valley Fever, and Monkeypox), Helminths (Haematobium infection, Onchocerca volvulus, Echinococcus granulosus, Trichuriasis, Soil transmitted helminths, Hookworm disease, Ascariasis, Schistosomiasis), Protozoa (Cryptosporidium, Toxoplasma gondii, Malaria, and Leishmaniasis) and Bacteria (Escherichia coli enteritis, Cholera, Pneumococcus, Salmonella, Leprosy, Helicobacter pylori, Streptococcus pyogenes, Staphylococcus aureus, Mycobacterium tuberculosis, Chlamydia trachomatis, Brucella, Wound infection). This systematic categorization facilitated focused insights into pathogen-specific disease burdens while maintaining a structured approach to analysing data from diverse sources. The use of standardized definitions for age groups further enhanced the consistency and reliability of the extracted data, enabling a more coherent interpretation of results across studies.

### Map production and visualization

The geographic distribution of NTDs in Yemen was analyzed using spatial mapping techniques to visualize the prevalence and burden of different disease categories. The maps were created using ArcGIS version 10.8.2, a geographic information system software widely utilized for spatial analysis and visualization [21]. The base layers for the maps were sourced from Geotag, which provides open-source geographic data, ensuring accuracy and accessibility. For this analysis, the included articles were geotagged based on the administrative divisions (governorates) of Yemen reported in the studies. Each article’s reported pathogens and diseases were categorized into four main groups: viral, bacterial, helminthic, and protozoal. The data were then aggregated to calculate the proportion of articles reporting each disease category within each governorate. A color gradient was applied to the maps, with darker shades representing higher proportions of reported cases for each etiological category. This method provided a clear visual representation of disease distribution across Yemen.

### Analysis and assessment of risk of bias for diagnosis ascertainment

The included studies exhibited significant heterogeneity across multiple dimensions, including study design, population characteristics, settings, sample types, diagnostic methods, and the depth of information provided about individuals tested. Examples of this variability included differences in diagnostic techniques, such as the use of microscopy versus molecular methods, disparities in target populations (e.g., displaced populations versus general community samples), and diverse study settings ranging from urban centers to remote rural regions with limited infrastructure. These inconsistencies precluded the use of meta-analysis to estimate pooled incidence and prevalence.

To address these challenges, the QUADAS-2 tool was adapted for evaluating diagnostic bias. Studies were categorized as having either a “high” or “low” risk of bias based on predefined criteria. Key factors included the diagnostic methods employed, the quality of reporting, and sample handling protocols. Studies using standard methods (e.g., PCR or ELISA) recommended by the CDC or WHO were classified as ‘low risk,’ while those relying on less reliable techniques (e.g., non-standard microscopy or clinical diagnosis) were rated ‘high risk. Similarly, studies providing detailed, transparent descriptions of diagnostic protocols, including reagents, instruments, and positivity thresholds, were considered “low” risk, whereas those with incomplete or ambiguous reporting were deemed “high” risk. The quality of sample handling, including adherence to standardized collection, storage, and processing procedures, was another critical determinant in the risk classification process.

In addition to diagnostic ascertainment bias, the review acknowledges other potential biases that could influence the findings. Publication bias can result from underreporting studies with null or unfavorable results, especially in peer-reviewed literature. Similarly, selection bias could affect the inclusion of grey literature, as these sources often lack standardized peer-review processes, potentially leading to variability in methodological quality. Efforts were made to minimize these biases by critically appraising grey literature for methodological rigor and transparency. Furthermore, the inclusion of grey literature aimed to reduce geographic and contextual biases by capturing local perspectives and data underrepresented in peer-reviewed publications.

To ensure consistency and minimize subjective bias in the risk of bias assessment, two independent reviewers evaluated each study using the adapted QUADAS-2 framework. Discrepancies in classification were resolved through discussion or consultation with a third reviewer. Inter-rater reliability was measured using the kappa statistic, which indicated substantial agreement (κ > 0.75) between reviewers, thereby enhancing the robustness of the assessment process. This rigorous approach provided a nuanced evaluation of diagnostic reliability and addressed the limitations imposed by the heterogeneity of the included studies. By acknowledging and addressing these multiple dimensions of bias, the review ensures a more transparent and balanced interpretation of its findings.

### Limitation of data and generalizability

The diversity of included studies poses challenges to generalizing findings. Variability in study objectives and geographic coverage across Yemen resulted in uneven representation of certain regions and population groups. For instance, conflict-affected rural areas with limited infrastructure were often underrepresented, potentially skewing the analysis toward data from more accessible regions. This uneven distribution limits the applicability of findings to regions with differing socio-environmental and health conditions.

Additionally, the absence of longitudinal data in many studies limits the ability to capture temporal trends in disease transmission and intervention outcomes. Without standardized follow-up periods or consistent reporting frameworks, it is difficult to assess how changes in conflict dynamics or humanitarian responses influence the prevalence and spread of NTDs. While efforts were made to include grey literature to address some data gaps, the lack of methodological details in these sources further complicates efforts to evaluate data quality comprehensively. This creates uncertainties in the reliability of findings from non-peer-reviewed sources, particularly when they contribute unique insights unavailable from other data types. These limitations highlight the need for more comprehensive and standardized data collection frameworks in future research. By addressing gaps in geographic coverage, ensuring the inclusion of longitudinal studies, and establishing uniform reporting standards, future studies can improve the reliability and generalizability of evidence on NTDs in conflict-affected settings.

## Results

### Summary of included studies

Out of 3,652 initially identified articles, 230 met the inclusion criteria and were included in this review. The publication timeline revealed that the earliest article dated back to 1955, representing 1% (3) of the included articles. Notably, 13% (29) of the articles were published between 2000 and 2010, while the majority (86%, 199 articles) were published after 2010. This significant increase in publications after 2000 reflects the availability of data retrieved from the databases, highlighting a growing global research interest in NTDs over the past two decades. This trend may also be attributed to the escalating public health impact of NTDs in Yemen and international efforts to address these diseases in conflict settings.

### Study population and study settings

**Fig. 2:**
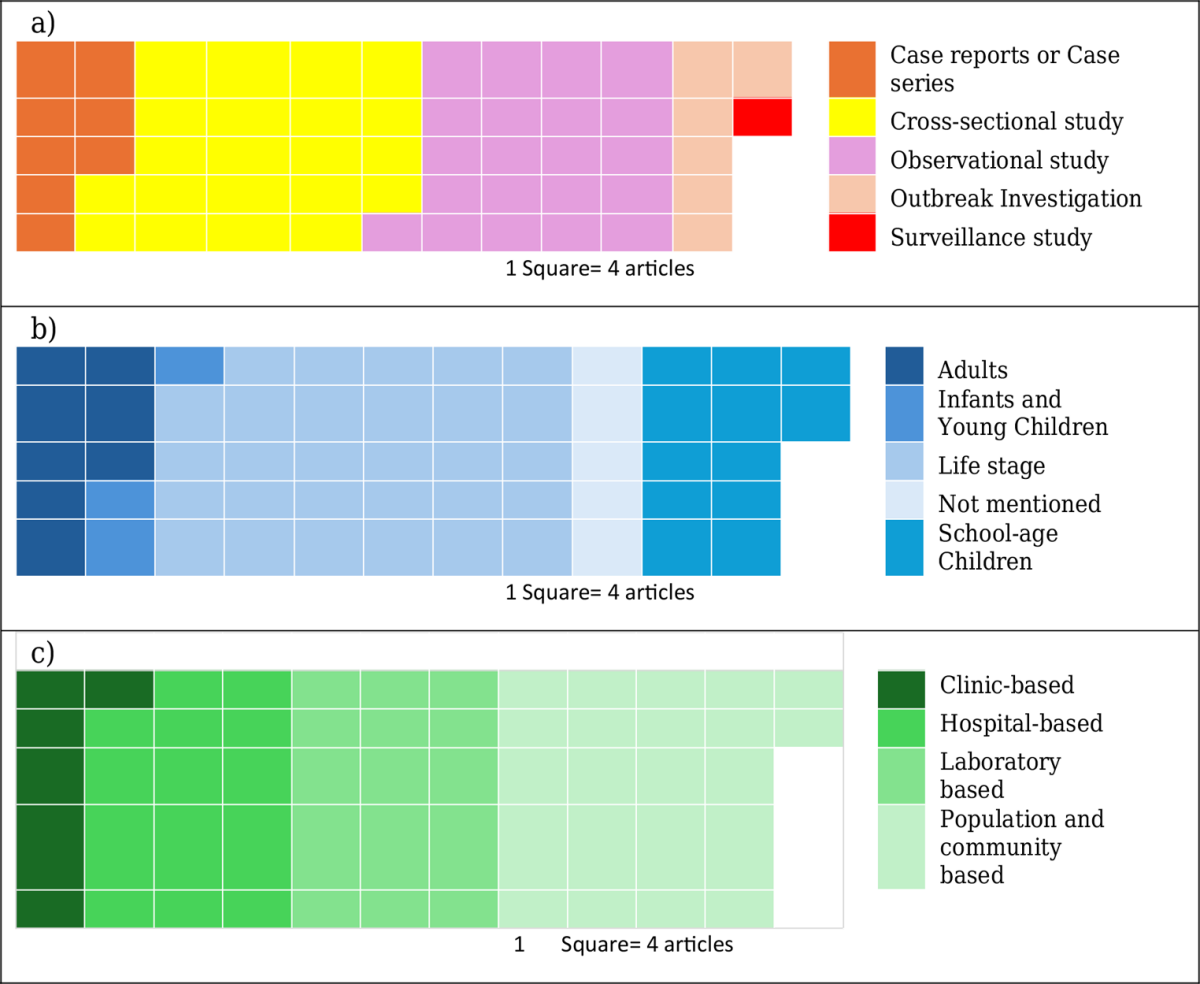
Waffle plots showing distribution of participant (**a**) study design, (**b**) age (study population), and (**c**) study setting of included articles

Among the included studies, the predominant design was observational (39%, 89 articles), followed closely by cross-sectional studies (36%, 83 articles). Case reports/series comprised 13% (31 articles), outbreak investigations 11% (25 articles), and surveillance studies were the least common at 1% (2 articles). This distribution underscores a reliance on observational and cross-sectional methodologies, which may limit insights into causation and longitudinal trends, highlighting the need for more robust study designs in future research (Fig. 2a).

Half of the studies (50%, 116 articles) examined life stages, encompassing all community age groups. Studies on school-age children comprised 22% (51 articles), while those focusing on adults represented 13% (31 articles). Notably, 4% (10 articles) focused on infants and young children, emphasizing the vulnerability of this age group to NTDs, particularly helminthic infections. The 10% (22 articles) that did not specify the age of participants indicate potential gaps in reporting and age-specific analysis (Fig. 2b).

Most studies were population- and community-based (39%, 89 articles), capturing broader epidemiological trends. Laboratory-based studies accounted for 27% (62 articles), while 24% (55 articles) were conducted in hospital settings. Clinic-based studies comprised 10% (24 articles), highlighting the varied contexts in which NTD research is conducted. These findings suggest that hospital- and clinic-based studies, which provide critical clinical data, remain underrepresented compared to broader community-level studies (Fig. 2c).

### Reported aetiology

**Fig. 3:**
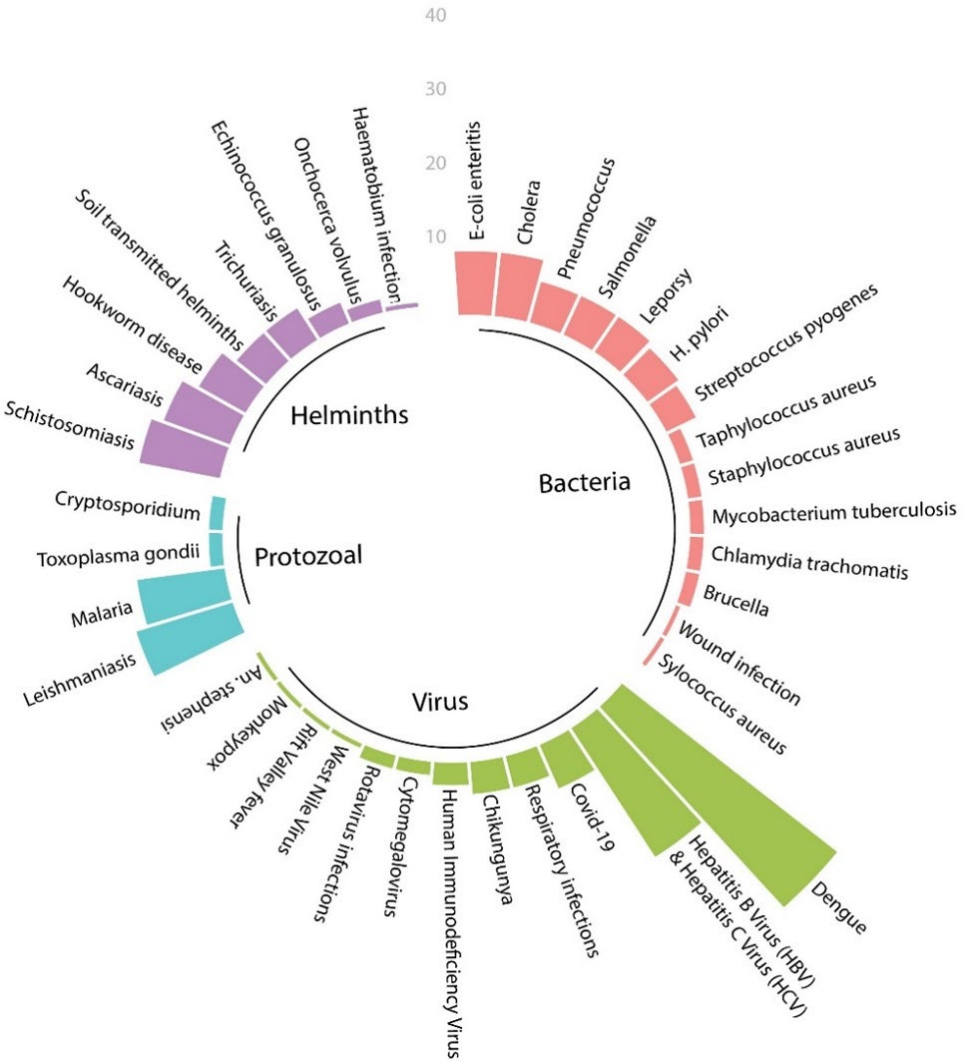
The circular bar plot categorizes reported NTDs into four main groups based on aetiology

Among the 230 articles, viral aetiology was most reported (39%, 89 articles), followed by bacterial (26%, 59 articles), helminthic (21%, 48 articles), and protozoal (15%, 34 articles). The dominance of viral and bacterial NTDs underscores the need for enhanced surveillance, vector control, and targeted interventions. (Fig. 3). Among bacterial NTDs, enteric bacteria such as *Escherichia coli*, *Cholera*, and *Salmonella* were most frequently reported, while arboviral diseases like dengue dominated viral NTD reports. Protozoal NTDs, particularly leishmaniasis and malaria, showed significant geographic clustering in central and western regions, while helminthic infections like schistosomiasis and soil-transmitted helminths disproportionately affected school-age children.

Bacterial NTDs: Of the 59 articles reporting bacterial NTDs, the most commonly mentioned were enteric bacteria, including *Escherichia coli*, *Vibrio cholerae*, and *Salmonella species*, appearing in 26 articles. This focus emphasizes the significant burden of gastrointestinal bacterial infections within the NTD context. Other bacterial diseases, such as leprosy caused by *Mycobacterium leprae*, infections due to *Streptococcus pyogenes*,* Helicobacter pylori*,* and Streptococcus pneumoniae* (Pneumococcus), were documented in 32 articles, covering a broad spectrum of bacterial infections. The diverse range of bacterial pathogens underscores the need for targeted interventions based on age-specific and region-specific disease patterns.

Viral NTDs: A total of 89 articles reported viral NTDs, with arboviral diseases being particularly prevalent. Notably, *Dengue virus* was the most frequently reported viral pathogen, appearing in 39 articles. The high frequency of *Dengue virus* underscores its significant public health impact, particularly in life-stage groups such as adults, and emphasizes the need for continuous surveillance and targeted vector control. Other arboviral pathogens reported included *Chikungunya virus*,* Monkeypox virus*,* Rift Valley fever virus*, and *West Nile virus*. Furthermore, other viral pathogens, such as *Hepatitis B Virus (HBV)*,* Hepatitis C Virus (HCV)*,* Human Immunodeficiency Virus (HIV)*,* Covid-19*, and various respiratory infections, were highlighted in the articles. The broad range of viral pathogens indicates the complexity of viral NTD epidemiology and calls for integrated viral surveillance and response strategies.

Protozoal NTDs: Four major protozoal NTDs were identified in 34 articles. Leishmaniasis emerged as the most commonly reported protozoal infection, with 16 articles, followed by malaria in 14 articles. The findings highlight the persistent burden of these two protozoal diseases in Yemen. Both leishmaniasis and malaria were more frequently reported among life-stage groups, indicating their widespread impact across all age categories. Other protozoal pathogens, such as *Toxoplasma gondii* and *Cryptosporidium parvum*, were also mentioned but less frequently, reflecting a narrower focus on these infections in the reviewed literature.

Helminth NTDs: Helminth infections were reported in 48 articles, with schistosomiasis caused by *Schistosoma mansoni and Schistosoma haematobium*, being the most frequently mentioned (13 articles). Ascariasis, caused by *Ascaris lumbricoides*, and hookworm infections, caused by *Ancylostoma duodenale* and *Necator americanus*, followed closely, appearing in 11 and 8 articles, respectively. These top three helminth infections were predominantly reported among school-age children, indicating a higher vulnerability of this age group to soil-transmitted helminth infections. Additionally, trichuriasis, caused by *Trichuris trichiura*, and other soil-transmitted helminths were reported in five articles each. Less frequently mentioned helminth infections included *Echinococcus granulosus*, *Onchocerca volvulus*, and *Schistosoma haematobium* infections, each reported in a few articles. These findings suggest the necessity of age-specific interventions, especially for school-age children, to reduce the burden of helminthiasis.

### Geographic location

**Fig. 4:**
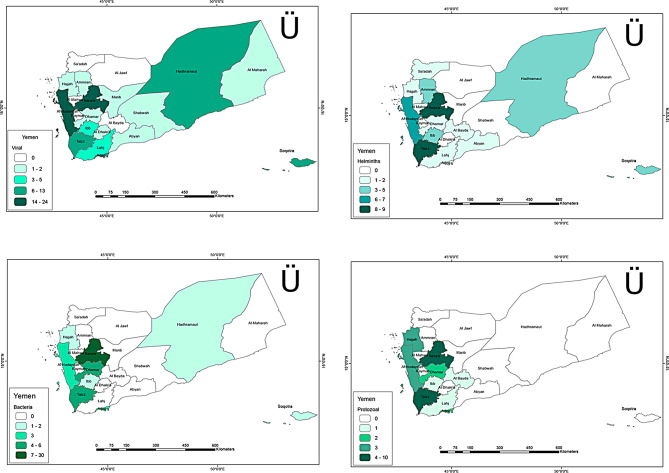
(**a**) Map showing location of reported Viral NTDs in the included articles, (**b**) Map showing location of reported Bacteria NTDs in the included articles. (**c**) Map showing location of reported Helminths NTDs in the included articles, (**d**) Map showing location of reported Bacteria NTDs in the included articles. The number inside each region represents number of pathogens reported in the articles and the colour represents percentage of total articles of corresponding aetiology. Maps were created using ArcGIS version 10.8.2 and the base layer of the map was produced using Geotag (https://geotag.sourceforge.net/)

The geographical distribution of reported NTDs, mapped using ArcGIS, revealed a concentration of cases in central and western regions, particularly Sana’a, Taiz, and Al Hudaydah (Fig. 4). These regions showed high prevalence of viral, bacterial, protozoal, and helminthic infections. This highlights the role of population density, poor water and sanitation infrastructure, and limited healthcare access in driving disease transmission. The consistent clustering across all aetiological groups highlights the urgent need for targeted public health interventions and infrastructure development in these regions.

## Discussion

This review highlights the aetiology, geographic distribution, and challenges of NTDs in Yemen, providing guidance for public health strategies. In terms of aetiology, viral NTDs were the most reported, followed by bacterial and helminthic NTDs, with protozoal NTDs being the least documented. Dengue Virus, HBV, and HCV were the most common viral pathogens. Dengue, in particular, was highlighted as a major public health concern, appearing in 39 articles. Its strong association with urbanization, population density, and the effectiveness of vector control measures underscores the urgency for integrated vector management programs [22–24]. Community-led source reduction and insecticide-treated materials, proven successful in similar settings, should be adapted and expanded in Yemen [25–29]. Bacterial NTDs, primarily caused by enteric pathogens like *Escherichia coli*, *Cholera*, and *Salmonella*, remain a significant public health challenge. These pathogens are strongly linked to deficiencies in water, sanitation, and hygiene (WASH) infrastructure, particularly in displaced populations [30–33]. Lessons from conflict-affected regions like South Sudan and Somalia illustrate that investing in decentralized WASH facilities and emergency water treatment programs can significantly reduce bacterial disease burdens. Yemen could adopt similar strategies to mitigate these risks [34].

Helminthic NTDs, including schistosomiasis and ascariasis, predominantly affected school-age children, reflecting the vulnerability of this demographic to soil-transmitted helminths [35, 36]. Community-based deworming programs have demonstrated success in reducing helminth prevalence in other low-resource settings, such as Kenya and Ethiopia. Such programs, combined with education on hygiene practices, could provide a sustainable approach for Yemen [37, 38]. Protozoal NTDs, though less reported, pose unique challenges due to diverse transmission routes. Leishmaniasis and malaria were the most reported protozoal diseases, with significant geographic disparities in prevalence. These findings emphasize the importance of targeted interventions, such as vector control for malaria and sandfly eradication for leishmaniasis. Additionally, there is a local study highlight the value of mobile health clinics in improving disease diagnosis and treatment in conflict-affected and remote areas [39, 40].

The geographic distribution of NTDs revealed a clear concentration of reported cases in northern Yemen, with Sana’a, Al Hudaydah, and Taiz accounting for the majority. While this concentration reflects higher research activity in these regions, it also underscores geographic disparities in NTD research coverage. Eastern and southern regions of Yemen remain underrepresented, likely due to logistical challenges and security concerns in conflict zones. Expanding research efforts to these areas is critical to understanding the full scope of NTD burden across Yemen. Efforts to address these gaps could benefit from collaboration with local organizations and leveraging remote technologies, such as satellite imaging and mobile data collection, to overcome access barriers.

The findings of this review have significant implications for public health strategies in Yemen. Prioritizing region-specific interventions, particularly in high-burden areas, is essential. For example, enhanced sanitation and water quality can reduce bacterial and helminthic NTDs. Integrated vector management programs are critical for addressing viral and protozoal diseases. Furthermore, capacity building in healthcare infrastructure, particularly in underrepresented and conflict-affected regions, should be a national priority. The review also highlights the importance of community engagement in disease prevention and control. Initiatives such as community-led WASH programs and education campaigns can foster local ownership and sustainability. International collaboration is indispensable for providing technical and financial support, particularly in Yemen’s ongoing conflict and humanitarian crisis. Examples of international partnerships, such as the Global Fund’s malaria control efforts in sub-Saharan Africa, can serve as models for scaling up NTD interventions in Yemen.

In addition, the “ONE HEALTH” initiative offers a critical framework for addressing NTDs, particularly in regions where human, animal, and environmental health are closely interlinked. By fostering interdisciplinary collaboration among public health professionals, veterinarians, environmental scientists, and policymakers, the ONE HEALTH approach can enhance the effectiveness of interventions [41, 42]. For example, zoonotic NTDs such as leishmaniasis and schistosomiasis require coordinated efforts to manage livestock health, control vectors, and improve water and sanitation systems. This initiative emphasizes the interconnectedness of ecosystems, advocating for integrated strategies to reduce disease transmission while considering the broader socioeconomic and ecological context in Yemen. By adopting the ONE HEALTH framework, stakeholders in Yemen can address the root causes of NTDs, fostering long-term resilience against future outbreaks. By addressing these gaps with a multifaceted approach that includes region-specific interventions, community-led initiatives, international partnerships, and the ONE HEALTH framework, Yemen can make significant progress in reducing the burden of NTDs. Ultimately, these efforts will play a crucial role in alleviating suffering, rebuilding public health infrastructure, and improving overall health outcomes in the country.

This review highlights several research priorities to address gaps in the current understanding of NTDs in Yemen. Future studies should: (1) Expand geographic coverage to include underrepresented regions, using innovative data collection methods such as satellite-based mapping and drone surveillance. (2) Focus on protozoal NTDs and underexplored areas like polyparasitism and co-infections, which remain poorly understood. (3) Incorporate longitudinal studies to assess the impact of interventions over time and capture temporal trends in disease transmission and (4) Evaluate the cost-effectiveness and scalability of successful interventions from other low-resource settings to inform policymaking in Yemen.

## Conclusion

This review provides a comprehensive understanding of NTDs burden and distribution in Yemen, revealing critical etiological patterns and geographic disparities. The findings underscore the need for multifaceted, region-specific interventions supported by strong political commitment and international collaboration. By addressing these challenges with innovative and sustainable strategies, Yemen can make significant strides in controlling and ultimately eliminating NTD.

## Data Availability

All relevant data are within the manuscript.
